# The adaptation and psychometric validation of a stigma measure for adults diagnosed with severe vision impairment in rural Mozambique

**DOI:** 10.1186/s40359-026-04542-1

**Published:** 2026-05-16

**Authors:** Stephen R Pye, Stevens Bechange, Emma Jolley, Mercia Cumaio, Abrão Banqueiro Chale, Valdemiro Bila, Delvina Pedro, MaiMai Jose Linha, Sancho Manuel Chivunde, Aurora Antunes, Ana Maria Tavares, Tesfaye Adera, Izidine Hassane, Anne Roca, Iain Jones, Anita Jeyam, Elena Schmidt

**Affiliations:** 1 Sightsavers United Kingdom, Haywards Heath, West Sussex UK; 2 Sightsavers Moçambique Country Office, Nampula, Moçambique; 3 Forum das Associações Moçambicanas de Pessoas com Deficiência, Nampula, Moçambique; 4 Hospital Central de Nampula, Nampula, Moçambique

**Keywords:** Africa, stigma, discrimination, cataract surgery, vision impairment, Mozambique

## Abstract

**Background:**

People living with severe visual impairment endure stigma and discrimination, which can contribute to poor health and economic outcomes. There are few tools that measure vision-related stigma in the populations of rural Africa. The aim of this study was to assess an adapted tool for measuring stigmatizing attitudes and behaviors among a cohort of adults with severe visual impairment in rural Mozambique.

**Methods:**

A cohort of 927 patients with severe visual impairment from rural Northern Mozambique were examined. As part of a wider study, an adapted stigma and discrimination tool consisting of 13 questions was administered to the cohort. 556 (60.0%) patients responded to all questions and were included in the subsequent analysis. The 556 patients were split into equivalent subsamples. An exploratory factor analysis (EFA) was conducted on the first subsample to examine the underlying constructs of the tool, followed by a confirmatory factor analysis (CFA) using the second subsample to validate the structure identified by the EFA.

**Results:**

Factor analysis of the tool revealed an underlying two-factor structure that mirrored the theoretical structure of the original tool. The models showed good fit based on standard model-fit indices and good internal consistency based on Cronbach’s alpha.

**Conclusions:**

The adapted tool showed good validity and reliability. These are encouraging results that can be progressed further to develop a standardized stigma and discrimination measurement among patients living with severe visual impairment in rural African settings.

**Trial registration:**

Clinical trial number: not applicable.

**Supplementary Information:**

The online version contains supplementary material available at 10.1186/s40359-026-04542-1.

## Background

Vision impairment (VI) is a major public health concern globally, with over 43 million people being blind and 295 million living with moderate to severe VI [1, 2]. Sub-Saharan Africa bears a disproportionate burden of VI from avoidable causes such as cataracts due to scarce human and financial resources, limited eye care infrastructure and low levels of awareness of eye diseases and services among the population [3].

Severe VI not only affects individuals’ functional abilities but also their psychosocial well-being. Studies have shown that individuals whose vision is severely impaired experience increased rates of depression and anxiety [4], reduced social participation [5], lower self-esteem and autonomy [6]. These outcomes are often caused and exacerbated by stigma, which can manifest in the form of verbal and physical abuse, neglect and social distancing [7, 8]. Several theoretical frameworks explain how stigmatization can hinder the quality of life, access to services, and community participation of those affected [9–11]. Recent research in southern Africa has documented how individuals with disabilities are marginalized or denied opportunities for social integration [12, 13]. Individuals living with blindness and severe VI are often labelled as a burden to others leading to their social isolation, low self-esteem and unwillingness to take up health and social services [14, 15].

Despite the known impact of VI-related stigma on health and social outcomes, there is a lack of culturally appropriate tools to assess its prevalence in sub-Saharan Africa settings. Existing stigma measures are often developed and validated for other disease conditions like HIV [16], epilepsy [17], and mental illness [18] and may not capture the nuanced experiences of stigma by people with severe VI, as VI affects primarily people in older age groups. As age is a significant determinant of how individuals interact with their social networks as well as the quantity and quality of such interactions, it is important to have valid and reliable instruments to adequately capture the disruption of such interactions due to stigma. Without such tools, it is difficult to quantify VI and evaluate interventions aiming to reduce it.

Mozambique is a country in the southeast of Africa with an estimated population of 35 million people. Despite being rich in natural resources, the country has one of the lowest positions in the global ranking on both Human Development Index (HDI) and Gross Domestic Product (GDP) per capita with over 82% of the population living below the international extreme poverty line of $3 a day [19]. Access to eye care services, particularly in rural parts of the country where over 62% of the population lives, is limited due to general poverty [20], insufficient number of eye care facilities and ophthalmic personnel [21, 22], and prejudices and socio-cultural beliefs surrounding disability [23]. Adapting and validating a stigma measure for adults with severe VI in rural Mozambique is essential for eye care programmes aiming to increase population access to essential services and thus eliminate avoidable blindness and VI.

This methodological work was an integral part of a study among a cohort of 927 adults with severe VI who were examined and referred for cataract surgery in eye care facilities of Nampula province in northern Mozambique in the period April to December 2023. The overall study aimed to assess general and vision-related quality of life, functional ability and the experience of stigma before and after the surgery. In this paper we focus on evaluating whether the tool used for measuring stigma demonstrated reliability and validity with patients with VI in this context.

## Methods

### Study participants and sampling

All patients were referred for cataract surgery through an eye care programme supported by the international non-governmental organisation Sightsavers. Participants were eligible for inclusion in the study if they were aged 18 years or older, residents of one of Nampula’s 23 districts, and diagnosed with operable cataract. A tool for assessing decisional capacity was developed based on The University of California, San Diego Brief Assessment of Capacity to Consent (UBACC) [24] and the MacArthur Competence Assessment Tool-Treatment (MacCAT-T) [25]. Individuals were excluded if, after three assessment attempts, they were not able to show sufficient understanding of consent or decisional capacity to answer survey questions of a personal nature due to severe cognitive impairments, were under 18, not resident in the province, or planning to relocate within 12 months, as follow-up was planned post-surgery as part of a broader study. All patients who met the above inclusion criteria and were referred for surgery in the study period were invited to participate.

### Measures and tools

Stigma was assessed using a stigma scale originally developed by Stangl et al. to measure HIV-related stigma in Zambia and South Africa [26]. Following a review of available stigma scales, the study team reached consensus that the items in the Stang et al. scale were most appropriate for adaptation to the context of vision impairment. The scale’s structure and item content aligned well with the dimensions of stigma relevant to this population, providing a suitable foundation for modification while maintaining conceptual validity. During adaptation for this study, one item, concerning disclosure of HIV status without permission, was removed as it was deemed irrelevant to individuals with severe VI. Four context-specific items were added, addressing concerns such as losing housing, losing customers, losing a potential sexual partner, and feeling discouraged from undergoing cataract surgery. To reduce recall bias, the reference period was shortened from 12 months to 3 months.

The tool included 13 questions asking participants about their experiences of stigmatizing attitudes and behaviors in the past three months. Participants were asked to respond using a four-point Likert scale (‘strongly agree,’ ‘agree,’ ‘disagree,’ ‘strongly disagree’) and were also able to respond ‘don’t know’ or could refuse to answer any question. The participants were also asked to report the frequency of their experiences: ‘never,’ ‘once,’ ‘a few times,’ ‘often.’. The included items are shown in Table 1, and the questionnaire is shown in supplemental Table 1. Stangl and colleagues devised the questions to capture two domains of stigma: questions 1–3 measured ‘internalized’ stigma, while questions 4–9 & 11–13 captured ‘experienced’ stigma [26]. An additional question (Q10) asked if the participant had confronted, challenged or educated someone about stigma. A domain was deemed present if the participant responded either ‘strongly agree’ or ‘agree’ to at least one question in that domain. The 4 item responses were coded 1–4 for ease of analysis.

**Table 1:** Items in stigma & discrimination tool

Items	Questions
1. I have lost respect or standing in the community 2. I think less of myself 3. I have felt ashamed 4. People have talked badly about me 5. I have been verbally insulted, harassed and/or threatened 6. I have been physically assaulted 7. I have felt that people have not wanted to sit next to me 8. Have lost housing or not able to rent housing* 9. Have been denied promotion or further training 10. Confronted, challenged or educated someone 11. Have lost a potential sexual partner* 12. Have been discouraged from going for cataract surgery* 13. Have lost customers*	4 response Likert scale (Strongly agree / agree / disagree / strongly disagree)
	4 response frequency of experiences (Never / once / a few times / often)

*additional questions specific to vision impairment

The broader study also collected data on patients’ socio-demographic characteristics, vision, functional difficulties and quality of life. A more detailed description of these measurements is reported elsewhere [27].

### Procedures

All scales and study procedures were pilot tested to ensure their suitability for the target population. The stigma scale was independently forward translated by a local consultant, and back translated from English into Portuguese and Emakuwa by members of the study team fluent in the three languages following standard guidelines for translation and cross-cultural adaptation of instruments [28, 29]. The data collection team consisted of four eye care professionals from Nampula Central Hospital and one representative from the Forum of Organizations of Persons with Disabilities (FAMOD). Team members received training in rapport-building techniques and methods for discussing sensitive topics. They conducted face-to-face structured interviews in a neutral and non-judgmental manner. The tools were administered electronically using tablets and the CommCare software [30]; each interview (including all used tools) lasted approximately 45 min. All interviews were conducted at the hospital prior to cataract surgery.

### Data analysis

Statistical analyses were conducted using Stata version 18 (www.stata.com) and R version 4.4.0 [31]. Descriptive statistics were used to examine the responses to the stigma tool and the characteristics of the cohort. To examine the influence of non-response, a subject was classified as a non-responder if they refused to answer or answered ‘don’t know’ to at least one question of the stigma scale. Chi-squared and logistic regression models were used to explore the differences between responders and non-responders with respect to key characteristics.

Exploratory factor analysis (EFA) was used to explore the underlying factor structure of the stigma tool followed by confirmatory factor analysis (CFA) to cross-validate this factor structure. For this purpose, we generate two equivalent subsamples using the SOLOMON method [32]. We conducted EFA on the first subsample and CFA on the second. The EFA and CFA were conducted on a complete case basis, i.e. only on participants who responded to all items. As the items were ordinal, factor analyses were based on the polychoric correlations [33].

EFA was implemented using the Stata command factormat (www.stata.com). Bartlett’s test of sphericity was employed to assess if the data is suitable for factor analysis, a significant result indicating the presence of significant correlations between the variables under consideration. The Kaiser-Meyer-Olkin (KMO) measure of sampling adequacy (MSA) was also used to assess the degree of intercorrelations between the items, where values above 0.7 were considered adequate and values under 0.5 unacceptable [34, 35]. The EFA was performed based on the polychoric correlations between the items to account for the ordinal nature of the data. The iterated principal factor method was used with an oblique rotation, permitting correlation between factors [33]. The choice of factors was determined using a mixture of objective (parallel analysis and Cattel’s scree plot) and theoretical criteria [33]. The simplest solution was chosen that made theoretical sense and fulfilled the following criteria: all factors were loaded by at least three items, items with complex loadings were dropped, factors are theoretically meaningful and demonstrate good internal consistency, with Cronbach’s alpha ≥ 0.7. A factor loading was determined to be important if the loading was ≥ 0.30 [34].

To assess the goodness-of-fit of the factor structure chosen following the EFA, CFA was conducted using R packages lavaan v0.6.19 [36] and semPlot v1.1.6 [37]. The WLSMV (weighted least square mean- and variance-adjusted) estimator was used as the data were ordinal [38]. The following indices were used to evaluate goodness-of-fit: RMSEA (root mean square error of approximation) < 0.05, TLI (Tucker-Lewis index) > 0.95, CFI (comparative fit index) > 0.95 and SRMR (standardized root mean square residual) < 0.08 [39]. Robust goodness-of-fit indices were calculated and reported to take into account the ordinal nature of the data. The standardized results of the CFA are illustrated using Structural Equation Modelling (SEM) graphs [40], with rectangles representing the observed variables, ovals representing latent variables (factors), unidirectional arrows representing the factor loadings and two headed-arrows representing the variances/covariances.

## Results

### Participant characteristics

Overall, there were 927 participants in the study. The frequency of responses and summary statistics (among the responders) for all 13 items in the stigma tool are shown in Table 2. 556 (60.0%) participants responded (strongly disagree / disagree / agree / strongly agree) to all 13 items, with 371 (40.0%) answering either ‘don’t know’ or refusing to answer at least one question. Q4 had the greatest proportion of ‘don’t know’ responses (20.7%). 15 participants (1.6%) refused to answer one item.

**Table 2:** Frequency of responses and summary statistics for the 13 items of the stigma tool

Item	Frequency of responses (%)						Summary statistics (among responders)			
	Strongly disagree (1)	Disagree (2)	Agree (3)	Strongly agree (4)	Don’t know	Refused to answer	Mean	SD	Skewness	Kurtosis
Q1	17.4	54.2	4.7	11.4	12.3	0.0	1.1	0.9	1.0	3.5
Q2	10.8	60.0	11.4	15.0	2.7	0.1	1.3	0.9	0.8	2.9
Q3	8.1	42.6	10.7	37.9	0.8	0.0	1.8	1.0	0.0	1.5
Q4	10.5	40.7	7.4	20.7	20.7	0.0	1.5	1.0	0.4	1.9
Q5	29.2	55.5	4.9	7.2	3.2	0.0	0.9	0.8	1.1	4.2
Q6	44.1	54.8	0.0	1.0	0.0	0.1	0.6	0.6	0.6	4.4
Q7	23.3	60.8	0.3	0.4	15.1	0.0	0.7	0.5	-0.3	4.3
Q8	33.7	64.9	0.3	1.1	0.0	0.0	0.7	0.5	0.3	4.9
Q9	35.8	62.8	0.1	1.2	0.1	0.0	0.7	0.5	0.4	5.0
Q10	22.8	73.7	1.1	2.0	0.4	0.0	0.8	0.5	0.7	7.0
Q11	27.1	69.3	0.4	2.2	0.0	1.0	0.8	0.6	0.7	6.5
Q12	23.6	64.1	3.1	9.2	0.0	0.0	1.0	0.8	1.1	4.5
Q13	27.3	70.1	0.6	1.3	0.3	0.4	0.8	0.5	0.3	5.8

*SD* = standard deviation

The characteristics of the 927 participants stratified by response are shown in Table 3. Among the responders, there were slightly more males than females (53.8% vs. 46.2%) and the majority were aged 60 and over (89.2%). 55.0% were either married or co-habiting, 75.5% stated farming as their main occupation, and 75.7% received no schooling. With respect to eye health, 36.5% were found to be blind on clinical assessment and 75.3% reported difficulty with seeing.

Compared to non-responders, responders had better visual acuity, less severe functional difficulty across all domains of functioning, reported worse general health and attended religious services more regularly. These characteristics were statistically significant (*p* < 0.05).

Overall, out of the 927 participants in the study, 59.0% reported experiencing at least one type of stigmatizing attitude or behavior in the three months preceding the study.

**Table 3:** Participant characteristics

	Responder *N* = 556		Non-responder *N* = 371		Overall *N* = 927	
	n*	(%)	n*	(%)	n*	(%)
Sex						
Male	299	(53.8%)	178	(48.0%)	477	(51.5%)
Female	257	(46.2%)	193	(52.0%)	450	(48.5%)
Age (years)						
20–49	20	(3.6%)	19	(5.1%)	39	(4.2%)
50–59	40	(7.2%)	17	(4.6%)	57	(6.2%)
60–69	166	(30.0%)	114	(30.7%)	280	(30.3%)
70–79	218	(39.4%)	136	(36.7%)	354	(38.3%)
80+	110	(19.9%)	85	(22.9%)	195	(21.1%)
Frequency of religious attendance						
Regularly	408	(73.4%)	168	(45.3%)	576	(62.1%)
Rarely / few	124	(22.3%)	166	(44.7%)	290	(31.3%)
Never	24	(4.3%)	37	(10.0%)	61	(6.6%)
Marital status						
Married / co-habiting	306	(55.0%)	194	(52.3%)	500	(53.9%)
Divorced / separated	39	(7.0%)	30	(8.1%)	69	(7.4%)
Widowed	164	(29.5%)	100	(27.0%)	264	(28.5%)
Single	47	(8.5%)	47	(12.7%)	94	(10.1%)
Occupation						
Farming	420	(75.5%)	294	(79.5%)	714	(77.1%)
Depends on others	110	(19.8%)	61	(16.5%)	171	(18.5%)
Other	26	(4.7%)	15	(4.1%)	41	(4.4%)
Education						
No schooling	420	(75.7%)	299	(80.6%)	719	(77.6%)
Primary / secondary	135	(24.3%)	72	(19.4%)	207	(22.4%)
Visual Acuity (best eye)						
Can see 6/12	68	(12.2%)	50	(13.5%)	118	(12.7%)
Early VI	36	(6.5%)	23	(6.2%)	59	(6.4%)
Moderate VI	129	(23.2%)	75	(20.2%)	204	(22.0%)
Severe VI	120	(21.6%)	38	(10.2%)	158	(17.0%)
Blind	203	(36.5%)	185	(49.9%)	388	(41.9%)
Functional difficulty (excluding sight)						
No	299	(53.8%)	93	(25.1%)	392	(42.3%)
Yes	257	(46.2%)	278	(74.9%)	535	(57.7%)
Vision difficulty						
No / some difficulty	131	(24.7%)	45	(12.3%)	176	(19.6%)
A lot / cannot do	400	(75.3%)	320	(87.7%)	720	(80.4%)
Vision-related general health						
Excellent / very good / good	64	(11.5%)	68	(18.3%)	132	(14.2%)
Fair	363	(65.3%)	223	(60.1%)	586	(63.2%)
Poor	129	(23.2%)	80	(21.6%)	209	(22.5%)
Daily anxiety or depression						
No	227	(61.5%)	341	(62.0%)	568	(61.8%)
Yes	142	(38.5%)	209	(38.0%)	351	(38.2%)

*the numbers for each characteristic won’t necessarily add up to the total N due to small amounts of missing data

### Factor analysis

**Fig. 1:**
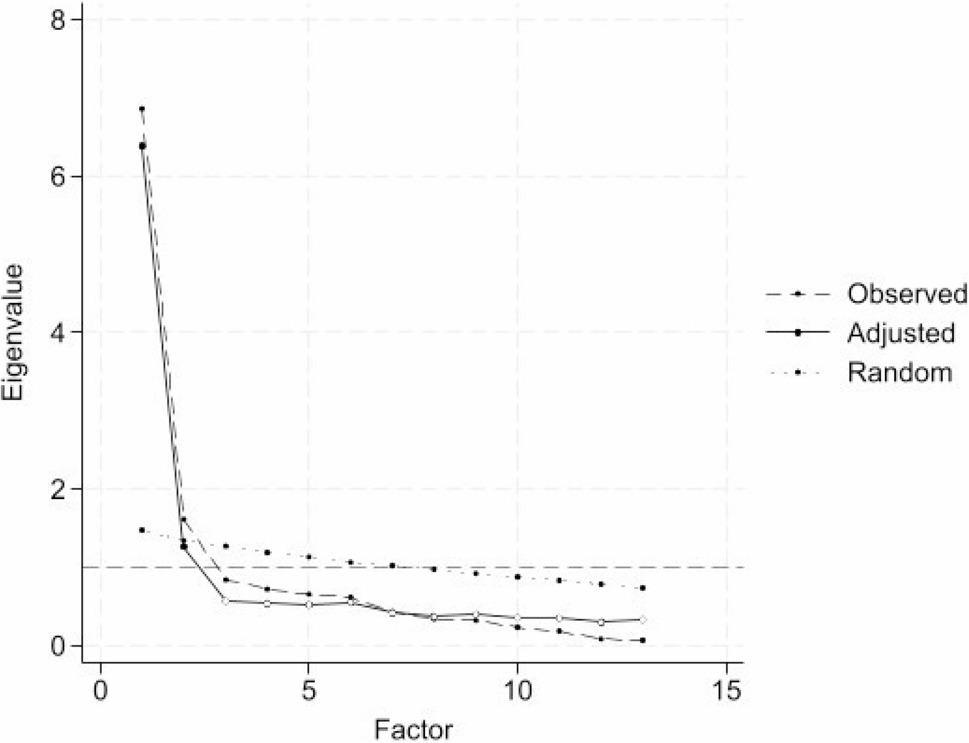
Parallel analysis showing the relationship between number of factors and eigenvalues for observed (study) and randomly generated data

The responses from the 556 participants that completed all 13 items of the stigma tool were taken forward into the factor analysis. The polychoric correlations between all 13 items are shown in supplemental Table 2. This dataset was then split into equivalent subsamples, generating a sample size of 278 for the EFA and CFA respectively. The responses to questions 1 through 13 of the stigma tool were found to be suitable for EFA based on Barlett’s test of sphericity (*p* < 0.001), an overall KMO-MSA value of 0.83, and individual item KMO-MSA values ranging from 0.77 to 0.92. The result from the parallel analysis is shown in Fig. 1. The observed (study) and randomly generated data intersected at 2 factors with an eigenvalue > 1. This result was consistent with the Scree Plot and met the criteria described in the methods section. The result explained 88.8% of the variance.

The factor loadings from the EFA are shown in Table 4. The following items loaded exclusively onto a first factor: being physically assaulted, feeling that people have not wanted to sit nearby, lost housing, denied promotion, confronted someone, lost a partner, discouraged from going for cataract surgery and lost customers. The following items loaded exclusively onto a second factor: lost respect, thinking less of myself, feeling ashamed and reporting that others have talked badly about me. One item cross-loaded onto both factors: being verbally assaulted. The two factors were correlated (0.55). Cronbach’s alpha was 0.85 (95%CI: 0.82–0.88) overall, and 0.83 (95%CI: 0.80–0.87) & 0.78 (95%CI: 0.74–0.83) for factors 1 and 2 respectively, indicating good internal consistency.

**Table 4:** EFA factor loadings on the agree / disagree Likert scale questions

Item	Factor 1	Factor 2
Q1. I have lost respect or standing in the community	0.04	0.62
Q2. I think less of myself	0.01	0.75
Q3. I have felt ashamed	0.11	0.64
Q4. People have talked badly about me	-0.11	0.87
Q5. I have been verbally insulted, harassed and/or threatened	0.35	0.52
Q6. I have been physically assaulted	0.80	0.12
Q7. I have felt that people have not wanted to sit next to me	0.81	-0.15
Q8. Have lost housing or not able to rent housing	0.86	0.03
Q9. Have been denied promotion or further training	0.71	0.20
Q10. Confronted, challenged or educated someone	0.57	0.18
Q11. Have lost a potential sexual partner	0.86	0.02
Q12. Have been discouraged from going for cataract surgery	0.59	0.07
Q13. Have lost customers	0.87	-0.05

**Fig. 2:**
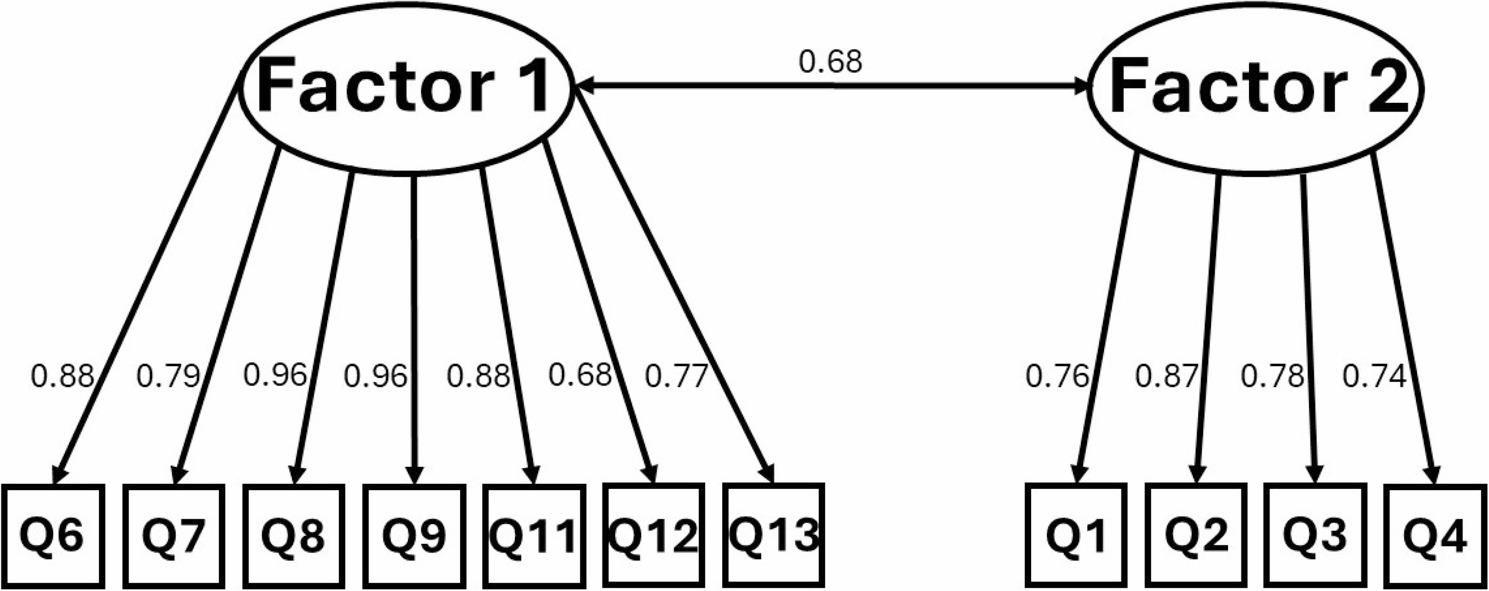
Results from the CFA

The results from the CFA are shown in Fig. 2. Q5 cross-loaded onto both factors in the EFA and so was excluded from the CFA. Q10 was also excluded as this was a stand-alone question in the original tool [26]. Factor 1 consisted of 7 of the questionnaire items (Qs 6–9 & 11–13), while factor 2 grouped around a further 4 items (Qs 1–4). The two-factor result demonstrated an adequate fit with the following indices: Chi-square = 74.099 on 43 degrees of freedom, CFI = 0.991, TLI = 0.988, RMSEA = 0.026, with the SRMR being just slightly above the acceptable range at 0.094. All factor loadings were statistically significant (*p* < 0.05). Cronbach’s alpha was 0.85 (95%CI: 0.82–0.88) overall, and 0.84 (95%CI: 0.81–0.88) & 0.79 (95%CI: 0.74–0.84) for factors 1 and 2 respectively, indicating good internal consistency, and very similar reliability results to those from the first subsample.

Preliminary convergent validity of the stigma tool was supported by findings from a baseline analysis of the same cohort presented elsewhere [27], which examined associations between stigma and theoretically related health measures. Higher internalized and experienced stigma were significantly associated with poorer self‑reported general health, greater self‑reported difficulty in seeing, worse objective visual acuity, and lower overall vision‑related quality of life. These associations align with the Health Stigma and Discrimination Framework [41], which emphasizes the relationship between declining health, reduced autonomy, and increased vulnerability to stigma across health conditions.

## Discussion

This study adapted and assessed a pre-existing tool developed to measure stigma and discrimination in HIV patients for use in subjects with vision impairment in a rural sub-Saharan African setting. The tool was found to be easy to use, demonstrated good internal consistency, and revealed a factor structure that mirrored the theoretical structure of the original stigma tool [26]. Our findings suggest that the adapted scale is a reliable and valid instrument for measuring stigmatizing attitudes and behaviors related to severe VI in rural Mozambique.

The baseline cohort analysis offers additional convergent validity evidence for the adapted stigma scale [27]. Internalized and experienced stigma were consistently associated with vision‑related difficulty, poorer measured visual acuity, lower vision‑related quality of life, and poorer general health status, as documented in the baseline analysis of this cohort. These findings are consistent with broader evidence showing that poorer physical health and functional limitations contribute to elevated experiences of stigma and its psychological consequences among people with disabilities and chronic health conditions [42, 43]. The close alignment between theoretical expectations, baseline cohort findings, and established stigma frameworks reinforces confidence in the construct validity of the tool.

Beyond demonstrating acceptable psychometric performance, our analysis advances understanding of vision‑related stigma in several important ways. First, the findings show that the experiences of adults with severe VI in rural Mozambique map clearly onto the dual structure of internalized and experienced stigma, consistent with the multidimensional structure established in HIV stigma research [26]. Evidence from disability‑related stigma reviews in sub‑Saharan Africa further underscores the importance of tools that can differentiate between multiple stigma dimensions, given the complex forms of exclusion documented among disabled populations [44]. Second, the adapted items capture forms of social exclusion relevant to visually impaired adults, such as avoidance, loss of customers, and discouragement from surgery, which are echoed in broader analyses of the social and economic marginalization of visually impaired people in African contexts [45]. These contributions represent an innovation in a field where validated, context‑specific stigma instruments for vision impairment are absent.

The adapted tool also has practical utility for eyecare staff, programme implementers, and researchers working in low‑resource settings. It aligns with the Health Stigma and Discrimination Framework, which emphasizes the need for standardized, actionable measures to guide programme design and stigma‑reduction strategies [41]. Its brevity and suitability for interviewer administration make it feasible for integration into pre‑surgical assessment workflows, community outreach, and follow‑up care. This is especially relevant in settings where disability‑related stigma is a recognized barrier to service use and requires systematic monitoring to inform interventions, as highlighted in regional reviews of stigma reduction efforts in Africa [44]. The high prevalence and health‑related correlates of stigma documented among this cohort further underscore its operational value in identifying individuals most vulnerable to stigma‑related barriers to cataract services.

Factor analysis of the tool revealed an underlying two-factor structure. The first factor represented 7 of the items (Qs 6, 7, 8, 9, 11, 12 & 13) and grouped similarly to the concept of ‘experienced’ stigma from the work of Stangl and colleagues [26]. The second factor represented a further 4 items (Qs 1–4) and grouped similarly to ‘internalized’ stigma from previous work [26]. In addition, one item (Q5) cross-loaded onto both factors and could therefore be considered as a stand-alone item in the tool. Q10, which related to the participant confronting, challenging or educating someone who was stigmatizing and/or discriminating against them, was also excluded from the CFA as it is considered a single item in the original tool [26].

To our knowledge, there are currently no other tools measuring stigma among patients diagnosed with severe vision impairment in rural African settings. The inclusion & exclusion criteria were carefully chosen to maximize the internal validity of the study. Despite the relatively small sample size, the tool developed here demonstrated good validity which also fit with theoretical criteria [26]. Stigma is common among people with VI across many countries in Africa, with many qualitative studies highlighting it as both an outcome of sight loss [46], and a cause, as it can create barriers to seeking eye health care [47, 48]. A reliable and valid tool that can help identify individuals at risk of different types of stigma is important to understanding those most at risk and identifying solutions to overcome the challenges they present.

Although several stigma instruments exist for health conditions such as HIV [16], mental illness [18], and epilepsy [17], few validated tools specifically represent the experiences of adults with vision impairment. Existing HIV stigma scales, such as those developed through the PopART trial [26], demonstrate robust multidimensional structures, but their content does not capture forms of economic exclusion or service‑related discouragement relevant to VI populations. Recent psychometric refinements of stigma instruments in HIV research also highlight the benefits of shorter, context‑adapted scales for programme use [49, 50], but these remain disease‑specific and do not address vision impairment. Systematic reviews of disability‑related stigma in Africa similarly conclude that available tools rarely address concrete behaviors such as avoidance, harassment, or loss of livelihood, which are frequently reported by adults with VI in African contexts [44]. The adapted instrument therefore fills a significant measurement gap by capturing both enacted and internalized forms of stigma that directly reflect the lived experiences of adults with severe VI.

Our study has a number of potential limitations. First, the study had a relatively small sample size, with only 556 subjects (60.0%) responding to all 13 items of the tool. With respect to non-response, it was not possible to analyze refusal and ‘don’t know’ responses separately due to the small numbers of subjects refusing to answer (*n* = 15). In addition to the descriptive comparison of responders vs. non-responders described in the results, the following differences between responders and non-responders remained statistically significant in a logistic model after adjustment for age and sex: religious attendance, and severe functional difficulty across all domains. Survey implementation conditions such as bystanders can increase the likelihood of non-response, and although we did not document bystanders here, the data were collected in busy hospitals which may have influenced the high proportion of ‘don’t knows’ [51]. Further work is needed to understand the reasons for non-response so that the response rate of the tool can be improved. The study was a facility-based study and stigma was measured among patients, who had made their way to hospital for surgery. These patients may be different from those who decided not to come for surgery, including their feelings and experiences of stigma. As patients were interviewed in a health facility, their responses may have been affected by desirability bias. Also, we conducted this study with a sample of patients targeted by a development eye health project. Although our respondents were from different districts throughout the province, the sample cannot be considered representative, which may limit the generalizability of our findings. In particular, all our participants were older adults with cataract. Our results cannot and should not be applied to younger population groups or people with other eye health conditions. Finally, the study was set in rural northern Mozambique and may not be applicable to other settings. While the study provides evidence of structural validity, internal consistency, and preliminary convergent validity, further research is required to assess divergent validity, test–retest reliability, and predictive validity in line with recommendations for robust stigma measurement frameworks.

## Conclusions

This study has shown promising results in adapting an HIV / AIDS stigma tool to measure stigma & discrimination in the context of patients diagnosed with severe vision impairment and has provided evidence that the tool shows good validity. Further research is needed to make more general recommendations regarding the content and adequate settings for these stigma measurements. Further research is also needed to expand validation of the tool, including divergent and predictive validity, as recommended in systematic analyses of disability stigma measurement gaps in African settings [44].

## Supplementary Information

Supplementary Material 1.

Supplementary Material 2.

## Data Availability

The datasets generated and/or analyzed during the current study are not publicly available due to restraints in the ethical approvals, but we are willing to collaborate upon request to the corresponding author.

## Abbreviations

AIDS: Acquired Immunodeficiency Syndrome
CFA: Confirmatory Factor Analysis
CFI: Comparative Fit Index
EFA: Exploratory Factor Analysis
FAMOD: Forum of Organizations of Persons with Disabilities
HIV: Human Immunodeficiency Virus
KMO: Kaiser-Meyer-Olkin
MSA: Measure of Sampling Adequacy
NGO: Non-governmental Organization
RMSEA: Root Mean Square Error of Approximation
SEM: Structural Equation Modelling
SRMR: Standardized Root Mean Square Residual
TLI: Tucker-Lewis Index
VA: Visual Acuity
VI: Vision Impairment
WHO: World Health Organization
WLSMV: Weighted Least Square Mean- and Variance- Adjusted
