# Supplementary material for: The adaptation and psychometric validation of a stigma measure for adults diagnosed with severe vision impairment in rural Mozambique
Source: BMC Psychol. 2026 May 16;14:1014. doi: 10.1186/s40359-026-04542-1 (PMC13349088; doi:10.1186/s40359-026-04542-1)
Supplement: Supplementary file 1 — Supplementary Material 1. [file 40359_2026_4542_MOESM1_ESM.docx]

Supplemental table 1. Stigma and discrimination questionnaire (adapted from Stangl et al. 2020 (26))

| **In the past 3 months, I (*fill in action*) because of my impaired vision** *Circle one.* | | | | | **If ‘yes’, how often were you *(fill in action)* in the past 3 months because your impaired vision?** *Circle one.* | | | | **Since you had cataract surgery, do you think you are more likely or less likely to *(fill in action)*?** *Circle one.* | | | |  |
| --- | --- | --- | --- | --- | --- | --- | --- | --- | --- | --- | --- | --- | --- |
|  | **Strongly Agree** | **Agree** | **Disagree** | **Strongly Disagree** | **Never** | **Once** | **A few times** | **Often** | | **More likely** | **Same** | **Less likely** | |
| Have lost respect or standing in the community | 3 | 2 | 1 | 0 |  |  |  |  | |  |  |  | |
| Think less of myself | 3 | 2 | 1 | 0 |  |  |  |  | |  |  |  | |
| Have felt ashamed | 3 | 2 | 1 | 0 |  |  |  |  | |  |  |  | |
| People have talked badly about me | 3 | 2 | 1 | 0 |  |  |  |  | |  |  |  | |
| Have been verbally insulted, harassed and/or threatened | 3 | 2 | 1 | 0 |  |  |  |  | |  |  |  | |
| Have been physically assaulted | 3 | 2 | 1 | 0 |  |  |  |  | |  |  |  | |
| Have felt that people have not wanted to sit next to me, for example, on public transport, at church | 3 | 2 | 1 | 0 |  |  |  |  | |  |  |  | |
| Have lost housing or not able to rent housing | 3 | 2 | 1 | 0 |  |  |  |  | |  |  |  | |
| Have been denied promotion or further training | 3 | 2 | 1 | 0 |  |  |  |  | |  |  |  | |
| Confronted, challenged, or educated someone who was stigmatising and/or discriminating against me | 3 | 2 | 1 | 0 |  |  |  |  | |  |  |  | |
| Have lost a potential sexual partner | 3 | 2 | 1 | 0 |  |  |  |  | |  |  |  | |
| Have been discouraged from going for cataract surgery | 3 | 2 | 1 | 0 |  |  |  |  | |  |  |  | |
| Have lost customers to buy my produce/goods or lost a job | 3 | 2 | 1 | 0 |  |  |  |  | |  |  |  | |
