# Supplementary material for: The adaptation and psychometric validation of a stigma measure for adults diagnosed with severe vision impairment in rural Mozambique
Source: BMC Psychol. 2026 May 16;14:1014. doi: 10.1186/s40359-026-04542-1 (PMC13349088; doi:10.1186/s40359-026-04542-1)
Supplement: Supplementary file 2 — Supplementary Material 2. [file 40359_2026_4542_MOESM2_ESM.docx]

Supplemental table 2. Polychoric correlations between the 13 items of the stigma tool

| Item | Q1 | Q2 | Q3 | Q4 | Q5 | Q6 | Q7 | Q8 | Q9 | Q10 | Q11 | Q12 | Q13 |
| --- | --- | --- | --- | --- | --- | --- | --- | --- | --- | --- | --- | --- | --- |
| Q1 | 1.00 |  |  |  |  |  |  |  |  |  |  |  |  |
| Q2 | 0.59 | 1.00 |  |  |  |  |  |  |  |  |  |  |  |
| Q3 | 0.43 | 0.61 | 1.00 |  |  |  |  |  |  |  |  |  |  |
| Q4 | 0.52 | 0.64 | 0.53 | 1.00 |  |  |  |  |  |  |  |  |  |
| Q5 | 0.51 | 0.54 | 0.53 | 0.68 | 1.00 |  |  |  |  |  |  |  |  |
| Q6 | 0.52 | 0.44 | 0.46 | 0.45 | 0.67 | 1.00 |  |  |  |  |  |  |  |
| Q7 | 0.29 | 0.30 | 0.25 | 0.25 | 0.43 | 0.70 | 1.00 |  |  |  |  |  |  |
| Q8 | 0.36 | 0.45 | 0.50 | 0.39 | 0.59 | 0.84 | 0.75 | 1.00 |  |  |  |  |  |
| Q9 | 0.41 | 0.50 | 0.53 | 0.41 | 0.58 | 0.74 | 0.64 | 0.86 | 1.00 |  |  |  |  |
| Q10 | 0.38 | 0.39 | 0.47 | 0.43 | 0.56 | 0.64 | 0.51 | 0.68 | 0.73 | 1.00 |  |  |  |
| Q11 | 0.37 | 0.44 | 0.47 | 0.34 | 0.51 | 0.70 | 0.66 | 0.71 | 0.73 | 0.61 | 1.00 |  |  |
| Q12 | 0.22 | 0.39 | 0.37 | 0.36 | 0.41 | 0.49 | 0.46 | 0.61 | 0.53 | 0.52 | 0.59 | 1.00 |  |
| Q13 | 0.38 | 0.45 | 0.45 | 0.38 | 0.53 | 0.67 | 0.53 | 0.63 | 0.70 | 0.61 | 0.80 | 0.54 | 1.00 |
